# Similar 1-year tibial implant migration, but higher continuous migration with cementless fixation: a retrospective RSA study of 990 patients with moderate to severe knee osteoarthritis

**DOI:** 10.2340/17453674.2026.46493

**Published:** 2026-08-31

**Authors:** Johannes Marsay DAL, Karina N LINDE, Søren RYTTER, Daan KOPPENS, Mohammad Ali Saad KARIM, Maiken STILLING

**Affiliations:** 1 Department of Orthopaedics, Aarhus University Hospital, Aarhus; 2 Department of Clinical Medicine, Aarhus University, Aarhus, Denmark

## Abstract

**Background and purpose:**

Aseptic loosening is a major cause of revision surgery. The grade of osteoarthritis (OA) may influence tibial implant migration and the risk of aseptic loosening. We aimed to investigate the association between preoperative OA grade and tibial implant migration in cemented and cementless total and unicompartmental knee arthroplasty.

**Methods:**

We performed a retrospective analysis on a prospective clinical cohort of 990 patients (990 knees). Preoperative OA was graded with the Kellgren–Lawrence (KL) scale and categorized as severe (KL 4) or moderate (KL 1–3). Tibial implant migration was measured by radiostereometric analysis (RSA) at 1, 2, and 5 years. The primary outcome was the 1-year mean difference in maximum total point motion (MTPM) between OA groups. The secondary outcome was the difference between groups in the proportion of patients with continuous migration (MTPM > 0.2 mm between years 1 and 2). Analyses were adjusted for age, sex, and body mass index (BMI).

**Results:**

610 patients were classified with severe OA, and 380 patients were classified with moderate OA. Mean 1-year MTPM was similar between OA groups. In the cementless cohort (n = 651), continuous migration occurred in 83/360 (23%) of severe OA cases vs 26/195 (13%) of moderate OA cases. Hence, patients with severe OA and a cementless implant had a mean 73% higher risk of continuous migration. In the cemented cohort (n= 339), comparable results for continuous migration were found.

**Conclusion:**

Mean 1-year MTPM tibial implant migration values were similar across OA groups. However, patients with severe OA who received cementless implants exhibited a higher risk of continuous migration.

Knee osteoarthritis (OA) is increasingly prevalent, with increasing numbers of primary knee arthroplasty (KA) procedures performed in Denmark and worldwide [1]. According to the latest Danish Knee Arthroplasty Register report, 15,092 primary KAs and 1,531 revisions were performed between 2024 and 2025 [2]. Due to this increase in primary KAs, the number of revision procedures has also increased, with aseptic loosening accounting for 17% of KA revisions in Denmark [2]. Aseptic loosening and subsequent revision can be predicted by early tibial implant migration, as measured by radiostereometric analysis (RSA) [3,4].

Overall, factors that influence KA outcome can be divided into 3 categories: surgery-related, prosthesis-related, and patient-related factors. Prominent patient factors reported in the literature include low preoperative bone mineral density (BMD) [5], lack of bisphosphonate use [6], female sex [7], high body mass index (BMI) [8], and smoking [8]. Other proposed factors include tibial implant size and type [7,8], abnormal contact kinematics [9], leg alignment [10], and preoperative gait or muscle activation patterns [11].

To our knowledge, no prior studies have investigated the association between OA and tibial implant migration. The radiographic severity of knee OA may reflect periprosthetic bone morphology, thereby influencing tibial implant migration and the risk of aseptic loosening. Our primary aim was to evaluate the mean difference in maximum total point motion (MTPM) between the moderate and severe OA groups after 1 year in the 4 implant groups (cementless TKA, cementless UKA, cemented TKA, cemented UKA).

Our secondary aim was to evaluate the association between OA grade and continuous migration (MTPM of > 0.2 mm between the 1- and 2-year follow-up).

## Methods

### Study design

This retrospective analysis was conducted using a prospective clinical cohort of 990 patients from the AutoRSA database, which comprises 1,600 patients scheduled for primary KA at Aarhus University Hospital between 2014 and 2018. Patients underwent either medial unicompartmental knee arthroplasty (UKA) or total knee arthroplasty (TKA). Preoperatively, standard weightbearing knee radiographs were obtained for OA grading. Postoperatively, patients underwent RSA immediately after surgery and were followed up with RSA for up to 5 years. The study is reported in accordance with the STROBE guidelines [12].

### Patient eligibility

For inclusion, patients were required to have at least 2 RSA examinations: a baseline and a follow-up. Eligible patients were limited to those undergoing primary KA with implant types for which computer-aided design models were available. Excluded implant types were Avon patellofemoral prostheses, NexGen Wedged prosthesis, revision prostheses, and lateral unicompartmental implants. If patients had both knees operated on in the inclusion period, only the first operated knee was included.

### Kellgren–Lawrence grading

The grade of knee OA was evaluated on preoperative radiographs using the Kellgren–Lawrence (KL) OA grading system, which consists of 4 grades (KL 1 = doubtful, KL 2 = minimal, KL 3 = moderate, and KL 4 = severe) [13]. A KL score was assigned to both the medial and lateral compartment of each included knee, and the highest score was included in the subsequent analyses. The patients were dichotomized based on their KL score into moderate (KL 1–3) and severe (KL 4) OA groups. 3 observer groups contributed to the analyses. Observer 1 evaluated 406 radiographs in consensus with another rater [14], while Observers 2 (n = 292) and 3 (n = 292) were independent raters in the present study. Intrarater reliability was evaluated on a subset of the radiographs by comparing 2 grading sessions from the same observer, separated by a 14-day interval, on the same randomly selected patients (Observer 1, n = 49, Observer 2, n = 20, and Observer 3, n = 20). Interrater reliability was evaluated by pair-wise comparison of gradings between 2 observers on the same randomly selected patients (Observer 1, n = 9, Observers 2 and 3, n = 34).

### Surgical procedure

All patients underwent KA performed by experienced orthopedic surgeons (n = 5) at Aarhus University Hospital. Each procedure adhered to the relevant surgical protocols outlined in the user manuals provided by the respective implant manufacturers. The cohort received 1 of 6 different implant types, which are listed in Table 1 and visually presented in Supplementary Figure S1. The choice of implant system and stem configuration was based on the operating surgeon’s preference and implant availability, reflecting routine clinical practice rather than a predefined selection algorithm.

**Table 1:** Distribution of implant models

Implants	Fixation	n
Oxford Partial Knee, UKA **^a^**	Cementless	391
NexGen Complete Knee Solution Cruciate		
Retaining Trabecular Metal Monoblock		
Tibial Component, TKA **^a^**	Cementless	244
NexGen Complete Knee Solution Stemmed		
Precoat, TKA **^a^**	Cemented	165
Oxford Partial Knee, UKA **^a^**	Cemented	113
Triathlon Primary Total Knee System, TKA **^b^**	Cemented	46
Triathlon Primary Total Knee System, TKA **^b^**	Cementless	16
Vanguard Interlok Knee System, TKA **^a^**	Cemented	15

UKA: unicompartmental knee arthroplasty, TKA: total knee arthroplasty.

a Zimmer Biomet, Warsaw, IN, USA.

b Stryker, Portage, MI, USA.

Eligibility for cementless implants was determined by patient age (< 75 years) and bone quality, ascertained through preoperative radiographs and intraoperative evaluation by the operating surgeon. During surgery, between 3 and 8 tantalum markers (1.0 mm) were inserted in the proximal tibia by use of a bead-gun (Kulkanon, Wenbergs Finmek, Gunnilse, Sweden) to facilitate RSA for evaluation of implant migration.

Postoperatively, all patients underwent a standardized rehabilitation regimen, which was identical for both TKA and UKA. Patients were discharged on postoperative days 1–2 upon the fulfillment of established clinical and functional criteria.

### Radiostereometric analysis (RSA)

Guidelines as outlined by the International RSA Society were followed [15]. All patients were mobilized allowing full weightbearing before supine RSA baseline images were performed within 2 days post-surgery. Follow-up RSA images were obtained at baseline (mean 4.3 days, SD 6.8), 1 year (mean 1.1 years, SD 0.1), 2 years (mean 2.1, SD 0.1), and 5 years (mean 5.1 years, SD 0.2) postoperatively. All imaging procedures were performed using a standardized imaging protocol, a uniplanar calibration cage (Aarhus Box 19, Medis Specials, Leiden, The Netherlands), and the Adora RSA equipment (NRT X-RAY, Aarhus, Denmark). 2 experienced investigators analyzed the images using Model-Based RSA (version 4.2015 software) (RSAcore, Leiden, the Netherlands). For all analyses, baseline RSA images served as the reference and the calibration box’s coordinate system was employed. To assess RSA precision [15], double examination RSA images were obtained at the 1-year follow-up. Prior to statistical evaluation, implant MTPM data for all patients was visually inspected on scatterplots, and in the case of deviating migration patterns (n = 20), the RSA analyses were checked and optimized. Subsequently, all 20 patients were still included in our analyses. The maximum acceptable mean error for rigid-body fitting (ME) was set at 0.35 mm, and the recommended condition number (CN) threshold for knee arthroplasty according to RSA guidelines was 120 [15]. Implant migration was assessed using MTPM, total translation, total rotation, signed migrations, and continuous migration, defined as an MTPM increase greater than 0.2 mm between the 1- and 2-year follow-ups [16].

### RSA analyses and precision

**Figure 1:**
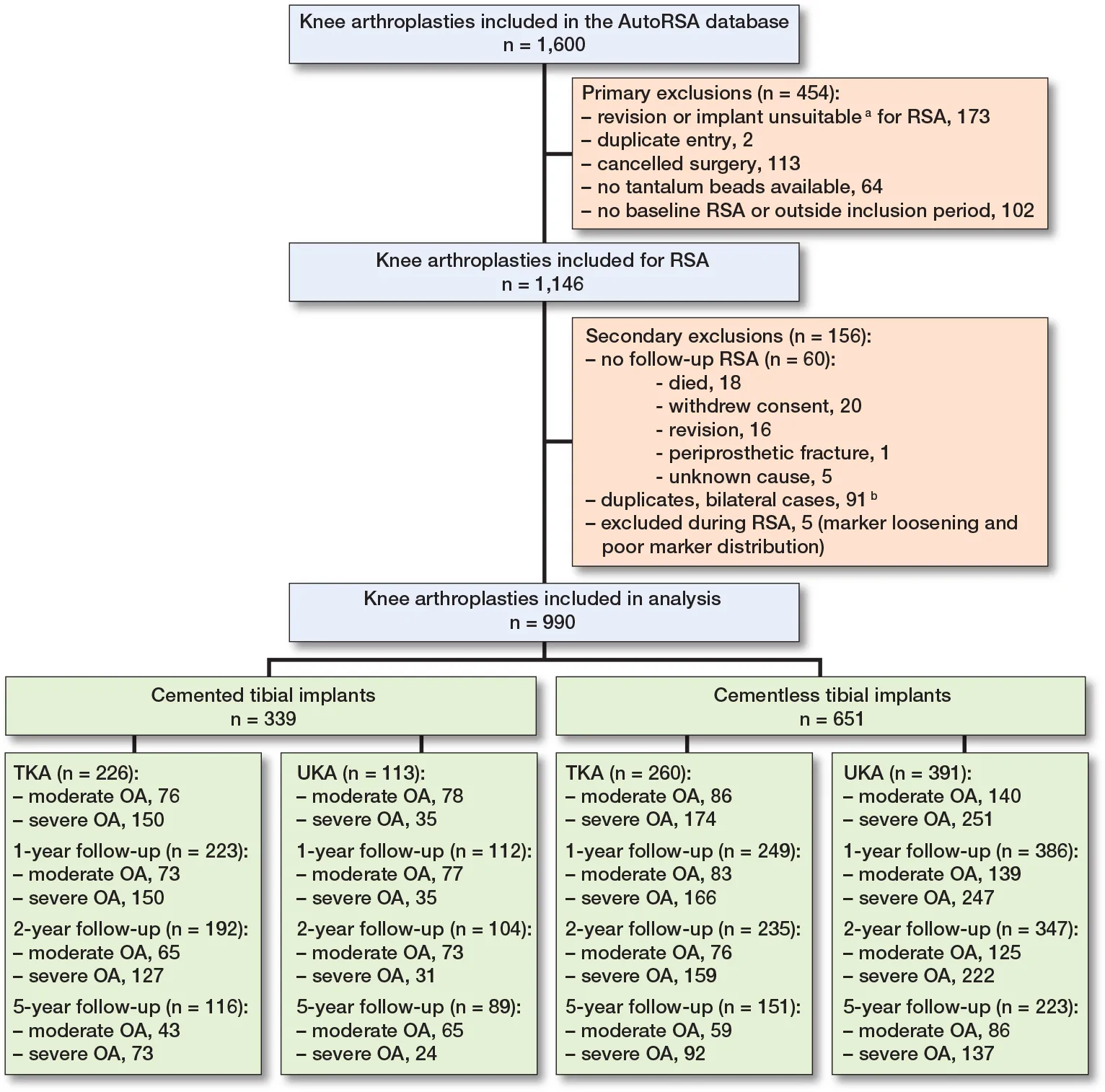
Flowchart of patients. RSA: radiostereometric analysis, TKA: total knee arthroplasty, UKA: unicompartmental knee arthroplasty. **^a^** No RSA model was available for these specific prosthesis models (Avon patellofemoral prostheses, Legacy Constrained Condylar Knee prostheses, NexGen Wedged, and lateral unicompartmental implants). **^b^** Duplicate entry due to bilateral surgery. Knee entered first was included.

5 patients were excluded from the final analysis due to technical errors during RSA analysis, which were caused by marker loosening and poor marker distribution (see Figure 1). The mean CN was 59 (SD 29, range 23–471). 27 patients had a CN above the recommended limit for knee arthroplasty of 120, and of these, 13 patients had a CN > 150. The 27 patients with a CN > 120 had a stable migration pattern, an acceptable mean ME (mean ME 0.22, SD 0.1), between 3 and 7 markers, and were included in the analysis. Sensitivity analyses excluding patients with CN > 120 did not alter the study conclusions. Mean 1-year ME for all patients was 0.18 (SD 0.1, range 0.0–0.4). RSA precision measured at 1-year follow-up (n = 882) is presented in Table 2.

**Table 2:** RSA double examination measurements at 1-year follow-up, specified for the 6 different implant types

Axis	Translations (mm)				Rotations (°)				MTPM (mm)
	x	y	z	TT ^a^	x	y	z	TR ^b^	
Oxford UKA (cementless) (n = 391)									
Mean difference	0.00	0.00	–0.00	0.01	–0.03	–0.01	0.02	0.01	0.01
SD difference	0.04	0.03	0.16	0.12	0.22	0.17	0.17	0.19	0.13
CR **^c^**	0.08	0.07	0.31	0.23	0.42	0.33	0.33	0.37	0.26
NexGen Trabecular Metal CR Monoblock (cementless) (n = 244)									
Mean difference	–0.00	0.00	0.00	–0.03	0.02	–0.05	–0.01	–0.08	–0.07
SD difference	0.08	0.06	0.31	0.20	0.37	0.30	0.14	0.30	0.22
CR **^c^**	0.16	0.13	0.62	0.39	0.72	0.58	0.28	0.59	0.44
NexGen Stemmed Precoat (cemented) (n =1 65)									
Mean difference	–0.00	–0.00	–0.02	0.01	–0.05	–0.05	0.00	–0.02	–0.02
SD difference	0.08	0.09	0.19	0.13	0.31	0.53	0.12	0.41	0.28
CR **^c^**	0.16	0.17	0.38	0.26	0.60	1.03	0.24	0.79	0.54
Oxford UKA (cemented) (n = 113)									
Mean difference	–0.01	–0.01	0.00	–0.01	0.02	–0.00	0.02	–0.01	–0.01
SD difference	0.05	0.03	0.11	0.07	0.21	0.19	0.16	0.16	0.09
CR **^c^**	0.10	0.07	0.21	0.14	0.40	0.36	0.32	0.31	0.18
Triathlon Primary TKA (cemented) (n = 46)									
Mean difference	–0.01	–0.01	0.01	–0.01	0.01	–0.04	0.02	0.01	–0.00
SD difference	0.05	0.04	0.14	0.10	0.26	0.28	0.09	0.24	0.19
CR **^c^**	0.10	0.08	0.27	0.20	0.52	0.54	0.18	0.48	0.37
Triathlon Primary TKA (cementless) (n = 16)									
Mean difference	–0.00	–0.03	–0.03	–0.00	0.08	0.05	0.01	–0.05	–0.02
SD difference	0.04	0.05	0.05	0.04	0.25	0.24	0.09	0.20	0.11
CR **^c^**	0.08	0.09	0.10	0.08	0.49	0.47	0.19	0.40	0.21
Vanguard Interlok CR with locking bar (cemented) (n = 15)									
Mean difference	–0.00	0.01	–0.01	–0.01	–0.01	0.03	0.03	–0.06	–0.05
SD difference	0.05	0.04	0.10	0.07	0.14	0.31	0.09	0.22	0.18
CR **^c^**	0.10	0.08	0.20	0.13	0.28	0.61	0.18	0.43	0.35

RSA: radiostereometric analysis, MTPM: maximum total point motion, TKA: total knee arthroplasty, UKA: unicompartmental knee arthroplasty, SD: standard deviation.

The mean difference represents the systematic measurement error. The SD difference represents the random variation (precision).

a Total translation (TT) was calculated using the Pythagorean theorem (TT= √(x2+y2+z2)).

b Total rotation (TR) was calculated using the Pythagorean theorem (TR= √(x2+y2+z2)).

c CR = coefficient of repeatability = 1.96. * SD difference represents the expected clinical precision.

### Intra- and interrater agreement

Weighted κ calculations for Kellgren–Lawrence grading of preoperative knee radiographs are given in Table 3.

**Table 3:** Intra- and interrater agreement (Cohen’s kappa, linear weighting) for Kellgren–Lawrence grading of preoperative knee radiographs

Analysis type Observer(s)	Patients, n	Cohen’s kappa (SE)	
		medial compartment	lateral compartment
Intrarater			
Observer 1	49	0.68 (0.11) **^a^**	0.63 (0.09) **^a^**
Observer 2	20	0.92 (0.17) **^b^**	0.86 (0.16) **^b^**
Observer 3	20	0.96 (0.17) **^b^**	0.79 (0.17) **^a^**
Interrater			
Observer 2 vs. 3	34	0.92 (0.14) **^b^**	0.82 (0.12) **^b^**
Observer 1 vs. 2 & 3	9	1.00 (0.00) **^b^**	0.80 (0.27) **^a^**

a Substantial agreement (κ = 0.61–0.80).

b Almost perfect agreement (κ = 0.81–1.00).

### Outcomes

The primary outcome was the mean difference in MTPM between the moderate and severe OA groups after 1 year in the 4 implant groups (cementless TKA, cementless UKA, cemented TKA, cemented UKA).

The secondary outcome examined the association between OA grade and continuous migration (MTPM of > 0.2 mm between the 1- and 2-year follow-up) in the 4 implant groups.

### Statistics

To analyze this, a mixed-effects repeated measurements model was applied with MTPM as the dependent variable. OA group, follow-up time (1, 2, and 5 years), and their interaction were included as fixed effects. Given the known differences in migration patterns between cemented and cementless fixation, analyses were stratified by implant type (TKA/UKA) and fixation method to account for this heterogeneity. Consequently, separate analyses were performed for the cemented TKA, cementless TKA, cemented UKA, and cementless UKA groups.

Follow-up time was modelled as a categorical fixed effect, and an interaction term between follow-up time and OA group was included to assess differences in migration trajectories over time. A directed acyclic graph (DAG) illustrating the assumed relationships between OA severity, tibial implant migration, and relevant covariates is provided in Figure S2 (see Supplementary data). Age, sex, and BMI were included as additional fixed-effect covariates, as these were the primary confounding variables available in our dataset. Both unadjusted and adjusted analyses were conducted. To account for within-patient correlation, a random intercept for patient was included, and an unstructured covariance matrix was used to model residual correlations across follow-up visits. Models were estimated using restricted maximum likelihood. Model-based marginal means (least-squares means) were estimated for each group at each follow-up time point, and between-group differences with 95% confidence intervals (CI) were derived from the fitted models. Missing data was assumed to be missing at random, and all available observations were included under the likelihood-based framework of the mixed-effects model. Inspection of Q–Q plots of residuals and plots of fitted values vs residuals supported the assumptions of linearity and normality.

In addition, differences between OA groups in total translation, total rotation, and signed migration were analyzed using mixed-effects repeated measurements models within each of the 4 implant groups across all follow-up time points, adjusted for age, sex, and BMI.

Binary regression was used to estimate risk ratios (RR), both unadjusted and adjusted for age, sex, and BMI. A sensitivity analysis was conducted, evaluating only patients with KL scores of 3 or 4, representing moderate and severe OA, respectively.

For comparison of baseline characteristics between OA groups, an unpaired two-sided Student’s t-test was used to compare continuous variables (age, BMI), and a chi-square test was used for proportions (sex). Normal data distribution was verified with probability plots.

Intra- and interrater reliability were assessed using Cohen’s weighted kappa (κ) with linear weights [17]. Kappa coefficients were presented with standard errors (SE).

All statistical analyses were performed using Stata 18.0 (StataCorp, College Station, TX, USA).

### Sample size

No sample size was calculated before the initiation of this specific study; however, an a priori calculation on minimal relevant differences based on power of 80% and significance level of 0.05 was calculated before statistical analyses were performed. The primary outcome measure was the MTPM tibial implant migration at 1 year in the 4 implants groups. We calculated the minimal detectable differences for each of the 4 implant groups using a power of 80% and a significance level of 0.05 (Table 4). The effect sizes were all considered clinically relevant.

**Table 4:** Specification of variables used for the estimation of detectable differences, including implant types, total sample size, unbalanced sample distribution between moderate and severe OA, standard deviation (SD) values with corresponding references, and the resulting minimal detectable differences

Implant type	Total, n	Moderate OA, n	Severe OA, n	SD **^a^** for MTPM (mm)	Detectable difference (mm)
Cementless TKA	260	86	174	0.7 **^b^**	0.26
Cementless UKA	391	140	251	0.6 **^c^**	0.18
Cemented TKA	226	76	150	0.4 **^b^**	0.16
Cemented UKA	113	78	35	0.3 **^c^**	0.27

MTPM: maximum total point motion, OA: osteoarthritis, SD: standard deviation, TKA: total knee arthroplasty, UKA: unicompartmental knee arthroplasty.

a SD values used for the estimation of the detectable difference were from RSA studies that reported SD values for mean MTPM (measured in mm) of tibial implant components at 12-month follow-up.

b For TKA, the study of Nakama et al. [26] was used.

c For UKA, we used data from an unpublished RSA study from our institution.

### Ethics, registration, data sharing plan, use of AI, funding and disclosures

Our study was approved as a quality assurance project under national regulations, for which informed oral consent was obtained and documented according to department procedures. Permission to publish without written consent from the AutoRSA database was obtained from the Danish National Ethics Committee and the Central Denmark Regions Committee (case number 2302161, issued March 27, 2023). The study was conducted in accordance with the Helsinki II declaration. The study was approved by the Danish Data Protection Agency (case number 1-16-02-54-14, issued on February 3, 2014). Data was available through the REDCap electronic data capture tools hosted at Aarhus University. Funding was received from Karen Elise Jensen’s Foundation, Health Research Foundation of Central Denmark Region, A.P. Moller Foundation, Danish National Advanced Technology Foundation, the Aarhus University Research Foundation, Orthopedic Research Foundation in Aarhus, the Development Fund at Aarhus University Hospital, and Aase and Ejnar Danielsens Foundation. The funding parties did not take part in the planning, execution, or interpretation of this study. During manuscript preparation, large language models (ChatGPT by OpenAI and Google Gemini) were used solely for language refinement and editorial suggestions. The authors critically reviewed and edited all AI-assisted text to ensure accuracy and originality. No AI tools were used for data generation, analysis, interpretation, or decision-making. The authors declare no conflict of interests. Complete disclosure of interest forms according to ICMJE are available on the article page, doi: 10.2340/17453674.2026.46493

## Results

### Patient characteristics

990 patients with cemented (n = 339) and cementless (n = 651) tibial implants (UKA and TKA) were evaluated during 5 years of prospective follow-up. The subgroupings (TKA and UKA) and OA severity grade have been outlined in Figure 1.

The study cohort included 610 patients with severe OA and 380 patients with moderate OA. Mean age was similar across fixation and procedure groups, except for cemented TKA where patients were older and there were significantly more women than men. BMI was overall similar across groups (Table 5). 76 patients were graded KL 1–2 and the indications for KA in this subgroup are presented in Table S2 (see Supplementary data). For all other patients, the indication for KA was primary or secondary OA, osteonecrosis, or inflammatory arthritis.

**Table 5:** Baseline characteristics stratified by implant type and osteoarthritis grade

Implant Factor	Moderate OA (n = 380)	Severe OA (n = 610)	Total
Cementless TKA, n	86	174	260
Age, mean (SD)	63 (9.1)	65 (8.6)	64 (8.8)
BMI, mean (SD)	28.1 (4.0)	30.0 (5.0)	29.4 (4.8)
Men/women, n	38/48	88/86	126/134
Cementless UKA, n	140	251	391
Age, mean (SD)	67 (9.3)	68 (8.9)	68 (9.1)
BMI, mean (SD)	28.9 (4.5)	28.4 (4.6)	28.6 (4.5)
Men/women, n	60/80	125/126	185/206
Cemented TKA, n	76	150	226
Age, mean (SD)	76 (7.1)	75 (10.5)	75 (9.5)
BMI, mean (SD)	27.6 (4.2)	28.9 (5.5)	28.5 (5.1)
Men/women, n	13/63	28/122	41/185
Cemented UKA, n	78	35	113
Age, mean (SD)	64 (8.9)	69 (8.9)	65 (9.2)
BMI, mean (SD)	29.0 (4.3)	27.7 (5.1)	28.6 (4.5)
Men/women, n	39/39	18/17	57/56

BMI: body mass index, OA: osteoarthritis, TKA: total knee arthroplasty, UKA: unicompartmental knee arthroplasty,

### Primary outcome

**Figure 2:**
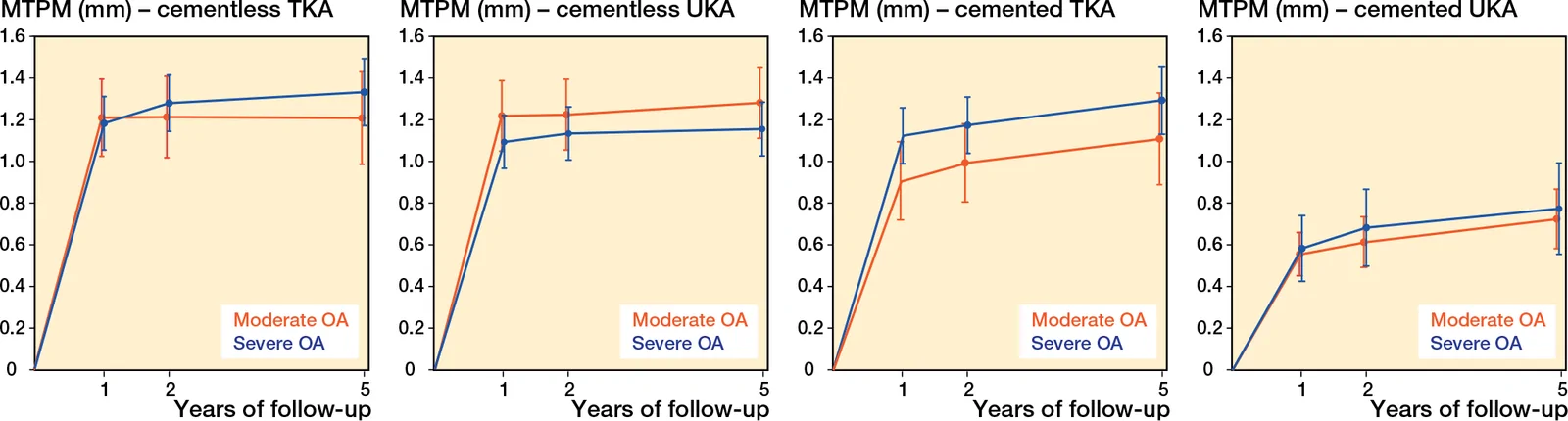
Mean MTPM with 95% confidence interval for moderate OA and severe OA groups across follow-ups.

The mean differences in 1-year MTPM between OA groups showed no difference for any of the 4 implant groups (Table 6, Figure 2). Primary outcome analyses were also performed stratified by implant type (Table S4, see Supplementary data). Unadjusted analyses yielded results that were numerically and qualitatively very similar to those of the adjusted models. Signed migrations, total translation, and total rotation are presented in Table S3A–D (see Supplementary data).

**Table 6:** Comparison of MTPM between OA groups presented in millimeters (mm) with corresponding mean (CI)

Implant Follow up	Moderate OA ^a^	Severe OA ^a^	Difference (severe – moderate)
Cementless TKA			
1-year	1.21 (1.03–1.39)	1.18 (1.06–1.31)	–0.03 (–0.25 to 0.20)
2-year	1.21 (1.02–1.41)	1.28 (1.15–1.42)	0.07 (–0.17 to 0.31)
5-year	1.21 (0.99–1.44)	1.33 (1.17–1.49)	0.12 (–0.15 to 0.40)
Cementless UKA			
1-year	1.22 (1.05–1.39)	1.09 (0.97–1.22)	–0.13 (–0.34 to 0.09)
2-year	1.23 (1.06–1.40)	1.13 (1.01–1.26)	–0.09 (–0.30 to 0.12)
5-year	1.28 (1.11–1.45)	1.16 (1.03–1.28)	–0.13 (–0.34 to 0.09)
Cemented TKA			
1-year	0.91 (0.72–1.09)	1.12 (0.99–1.25)	0.22 (–0.01 to 0.45)
2-year	0.99 (0.80–1.18)	1.17 (1.04–1.31)	0.18 (–0.05 to 0.41)
5-year	1.11 (0.89–1.33)	1.29 (1.13–1.45)	0.18 (–0.09 to 0.46)
Cemented UKA			
1-year	0.56 (0.45–0.66)	0.59 (0.43–0.74)	0.03 (–0.16 to 0.22)
2-year	0.62 (0.49–0.74)	0.68 (0.50–0.87)	0.07 (–0.15 to 0.29)
5-year	0.73 (0.58–0.87)	0.78 (0.56–0.99)	0.05 (–0.21 to 0.31)

For abbreviations, see Table 3 and MTPM: maximum total point motion, CI: 95% confidence interval

a Estimated mean MTPM in OA groups using mixed-effect model, adjusted for age, sex, and BMI.

### Secondary outcome

For cemented implants (TKAs and UKAs), comparable proportions of continuous migration were found in the moderate and severe OA groups. In the moderate OA group, 30 out of 134 patients (22%) demonstrated continuous migration, whereas the severe OA group had 40 out of 158 patients (25%) with continuous migration.

For cementless implants (TKAs and UKAs), the severe OA group had a higher RR of 1.73 (CI 1.15–2.59) for continuous migration compared with the moderate OA group. In the moderate OA group, 26 out of 195 patients (13%) demonstrated continuous migration, whilst the corresponding amount in the severe OA group was 83 out of 360 patients (23%). The corresponding unadjusted analysis without adjustment for age, sex, and BMI yielded an RR of 1.70 (CI 1.14–2.52).

Secondary outcome analyses were also performed stratified by implant type (Table S5, see Supplementary data).

### Sensitivity analysis

In the sensitivity analysis, patients with KL1 and KL2 were excluded, allowing a direct comparison between KL3 (moderate OA) and KL4 (severe OA). In patients with cementless implants (TKAs and UKAs) and OA grades KL3 (n = 186) and KL4 (n = 425), the sensitivity analysis revealed a higher risk of continuous migration in the severe OA group compared with the moderate OA group. The RR of continuous migration was 1.68 (CI 1.09–2.58) for KL4 patients compared with KL3 patients.

For patients with cemented implants, a similar sensitivity analysis showed comparable proportions of patients exhibiting continuous migration between severe and moderate OA groups.

## Discussion

This is the first study to investigate the association between OA grade and tibial implant migration. Prior research has largely focused on establishing the safety of various prosthesis types [18] and on identifying factors that may influence the implant failure risk. These factors include patient lifestyle [8], bone mineral density (BMD) [5], as well as mechanical parameters, such as preoperative gait patterns [19] and hip–knee angle [10].

We aimed to investigate the association between preoperative OA grade and tibial implant migration in cemented and cementless TKA and UKA. We found no differences in 1-year MTPM values between OA groups in both TKA and UKA. The secondary outcome analysis found no difference in continuous migration in OA groups for the cemented implants but a significantly higher proportion of continuous migration for the cementless implants, in patients with severe OA.

The observed differences between OA groups in the primary outcome analysis were close to the detectable differences. This suggests that the study was adequately powered with the available sample size, and the absence of statistically significant differences in the primary outcome may therefore reflect that the null hypothesis holds. However, residual confounding cannot be excluded, and small differences may become apparent in a larger cohort. Also, the substantial variability in migration results and patterns indicates that additional variables may influence the observed outcomes.

In our study, the 1-year MTPM for all implant groups in both OA groups was in the “at risk” category (0.54–1.6 mm) for 5-year tibial component revision [3]. In general, cementless implants exhibit higher initial migration; however, it is well established that cemented and cementless implants stabilize after the first year [20]. Therefore, continued tibial implant migration beyond 1 year, regardless of fixation method, serves as a surrogate marker for later aseptic loosening [16].

OA grade may serve as a surrogate for periprosthetic bone morphology, which could influence tibial implant migration and long-term outcomes. Accordingly, examining the association between OA severity and implant migration allows assessment of whether OA grade is a clinically relevant factor for implant fixation stability and implant survival.

The higher proportion of patients exhibiting continuous migration in the severe OA group may reflect a true association between OA grade and implant migration. Although our analyses were adjusted for principal risk factors for OA, such as age, sex, and BMI, residual confounding cannot be excluded. Patient data on hip–knee alignment [10] or genetic predispositions [21], which could also influence implant migration, were not available. Moreover, although we adjusted for age, the inherent comorbidities linked to higher age were not accounted for.

An explanatory factor of the association between severe OA and the increased risk of continuous migration in cementless implants, as found in this study, could be subchondral bone microarchitecture [5]. A recent study by Keiser et al. reported that in patients with advanced OA, the subchondral bone plate undergoes so-called “trabecular corticalisation,” marked by progressive sclerosis of the trabecular matrix [22]. These OA-related changes in bone morphology could plausibly affect the stability of both cemented and cementless tibial implants, potentially contributing to early implant migration. However, the relationship between OA-related alterations in bone morphology and early implant migration has not yet been investigated in KA. Studies have shown that patients with the most severe symptoms demonstrate the greatest improvement in patient-reported outcome measures (PROMs) after KA surgery [23,24]. Consequently, patient selection tends to favor those with the most severe OA. However, our data suggest that severe OA patients are also at higher risk of continuous implant migration—a known predictor of aseptic loosening and revision [3].

### Strengths

The major strength of this study lies in the employed RSA imaging method, which remains one of the most precise techniques available for measuring implant migration. Evaluation of RSA double examinations, CN, and ME demonstrated acceptable method reliability. Likewise, κ estimates indicated “substantial” to “perfect” agreement [25] and confirmed the reliability of the KL OA assessments. Another strength of the present study is the large number of patients included. In the intrarater analysis, the medial compartment κ values ranged from 0.68 to 0.96, whilst the lateral compartment showed κ values spanning from 0.63 to 0.86. In the interrater analysis, for the medial compartment κ values ranged from 0.92 to 1.00, whilst the lateral compartment showed κ values ranging from 0.80 to 0.82. Overall, our κ estimates indicated “substantial” to “perfect” agreement [25] and confirmed the reliability of the KL OA assessments.

### Limitations

Limitations of the study are the potential for residual confounding and the missing alignment data. The selection of fixation method was based on age and surgeon-assessed bone quality, which may have introduced selection bias. Furthermore, the KL grading system only assesses tibiofemoral OA in anteroposterior projection and therefore does not capture the presence or severity of OA seen in the lateral projection including patellofemoral OA, which may also influence patient symptoms and outcomes.

### Conclusion

Preoperative OA severity was not associated with mean 1-year MTPM migration. However, severe OA was identified as a risk factor for continuous migration in cementless implants.

*In perspective,* further studies are needed to investigate the association between subchondral bone microarchitecture and OA grade, as well as their effect on implant migration and risk of aseptic loosening.

### Supplementary data

Supplementary Tables S2–S5 and Figures S1 and S2 are available as supplementary data on the article page, doi: 10.2340/17453674.2026.46493

## Supplementary Material



## Notes

JMD: conceptualization, data curation, formal analysis, funding acquisition, methodology, project administration, validation, visualization, writing—original draft, writing—review and editing. KNL, MS: conceptualization, data curation, formal analysis, funding acquisition, investigation, methodology, project administration, resources, software, supervision, validation, writing—review and editing. SR: conceptualization, data curation, investigation, resources, supervision, writing—review and editing. DK: data curation, investigation, resources, writing—review and editing. MASK: formal analysis, visualization, writing—review and editing.

The authors thank all participating patients and the research staff at Aarhus University and Aarhus University Hospital for their support. Kjeld Søballe and Frank Madsen are acknowledged for their contributions to establishing the AutoRSA Database. The authors also thank Rikke Mørup for assistance with patient follow-up, DXA measurements, and RSA analyses, and Anne Dorthe Riedel for additional support with RSA analyses. The knee surgeons at Aarhus University Hospital are acknowledged for patient inclusion and surgical procedures.

Handling co-editors: Bart Pijls and Jonas Ranstam

*Acta* thanks Leif Ryd and other anonymous reviewers for help with peer review of this manuscript.
