# Supplementary material for: Similar 1-year tibial implant migration, but higher continuous migration with cementless fixation: a retrospective RSA study of 990 patients with moderate to severe knee osteoarthritis
Source: Acta Orthop. 2026 Aug 31;97:553–60. doi: 10.2340/17453674.2026.46493 (PMC13527607; doi:10.2340/17453674.2026.46493)
Supplement: Supplementary file 1 [file ActaO-97-46493-s1.pdf]

Supplementary data

Supplementary Figure S1. Visual representation of the specific prosthesis models included in the present study.

Oxford UKA[a], Cementless	NexGen TRAB MET CR Monoblock[b], Cementless	NexGen Stemmed Precoat[c], Cemented	Oxford UKA, Cemented[a]	Triathlon Primary TKA[d], Cemented and cementless	Vanguard TKA[e], Cemented
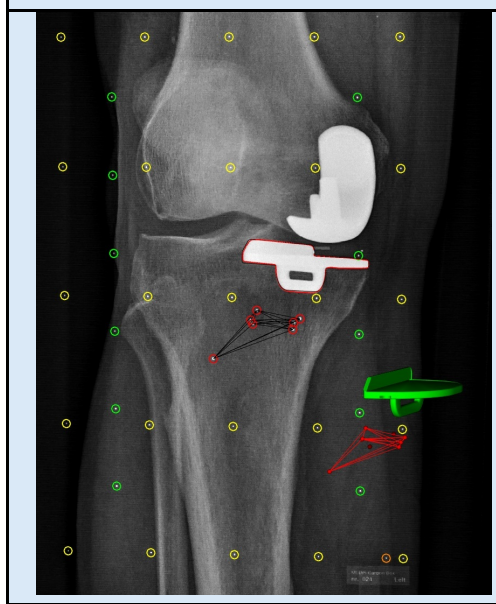	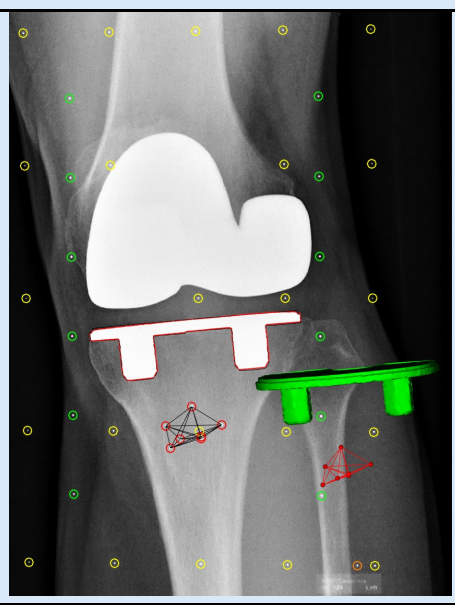	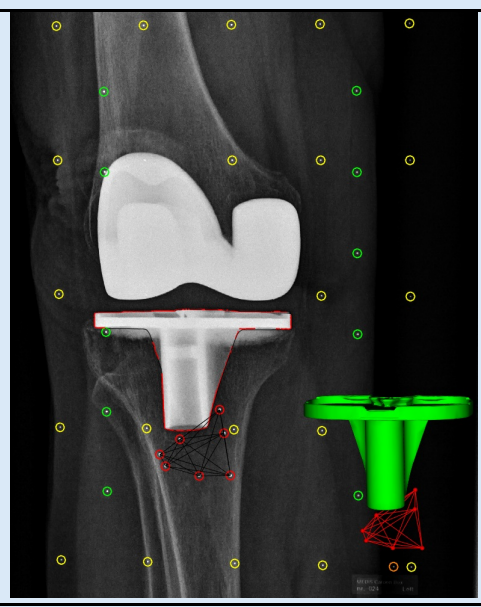	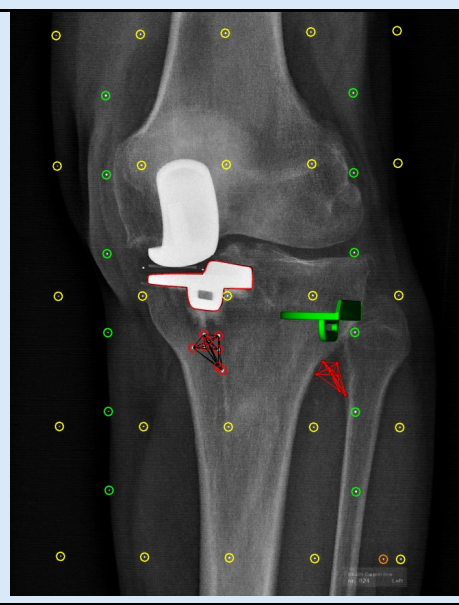	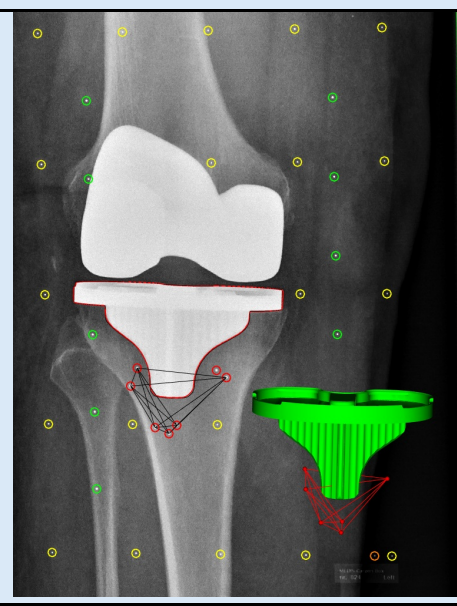	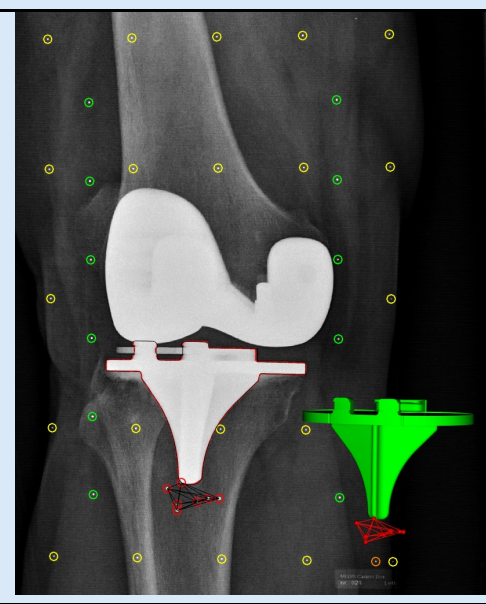

[a] Oxford® Partial Knee, [b] NexGen® Complete Knee Solution Cruciate Retaining (CR) Trabecular Metal™ Monoblock Tibial Component, [c] NexGen® Complete Knee Solution (stemmed, cemented tibial components), [d] Triathlon Primary Total Knee System (both cemented and cementless options), [e] Vanguard® Knee System (cemented TKA option)

Supplementary Table S2. Primary indications for arthroplasty among patients graded KL 1–2 in the femorotibial compartment (n = 76)

**Indication**	**n**	**(%)**
Isolated medial osteoarthritis	23	(30)
Patellofemoral osteoarthritis	29	(38)
Osteonecrosis	13	(17)
Rheumatoid arthritis	7	(9.2)
Prior patellectomy with instability	2	(2.6)
Osteochondritis	1	(1.3)
Subchondral cysts	1	(1.3)
**Total**	76	(100)

Supplementary Table S3a. Mean migration values in moderate- and severe OA groups across cemented and cementless implants with 95% confidence intervals (CI)

Follow-up	Moderate OA mm (CI)	Severe OA mm (CI)	Mean difference mm (CI)
**Cementless TKA**			
Mean Tx, mm			
1-year	-0.00 (-0.07 to 0.07)	0.01 (-0.04 to 0.06)	0.01 (-0.07 to 0.09)
2-year	0.00 (-0.06 to 0.07)	0.01 (-0.04 to 0.05)	0.01 (-0.08 to 0.09)
5-year	0.00 (-0.08 to 0.08)	0.00 (-0.06 to 0.06)	-0.00 (-0.10 to 0.10)
Mean Ty, mm			
1-year	-0.13 (-0.19 to -0.06)	-0.06 (-0.11 to -0.02)	0.06 (-0.01 to 0.14)
2-year	-0.12 (-0.18 to -0.05)	-0.05 (-0.09 to -0.00)	0.07 (-0.01 to 0.15)
5-year	-0.07 (-0.14 to -0.00)	-0.05 (-0.10 to 0.00)	0.02 (-0.07 to 0.11)
Mean Tz, mm			
1-year	-0.05 (-0.15 to 0.06)	-0.03 (-0.11 to 0.04)	0.01 (-0.12 to 0.15)
2-year	-0.07 (-0.18 to 0.4)	-0.09 (-0.16 to -0.01)	-0.02 (-0.15 to 0.12)
5-year	-0.06 (-0.19 to 0.06)	-0.07 (-0.16 to 0.03)	-0.00 (-0.16 to 0.15)
Mean TT, mm			
1-year	0.54 (0.45 to 0.62)	0.53 (0.48 to 0.59)	-0.00 (-0.11 to 0.10)
2-year	0.53 (0.45 to 0.62)	0.56 (0.50 to 0.62)	0.03 (-0.08 to 0.13)
5-year	0.54 (0.44 to 0.65)	0.61 (0.54 to 0.69)	0.07 (-0.06 to 0.20)
Mean Rx, °			
1-year	-0.06 (-0.36 to 0.24)	-0.12 (-0.33 to 0.09)	-0.06 (-0.43 to 0.31)
2-year	-0.17 (-0.48 to 0.15)	-0.13 (-0.35 to 0.08)	0.03 (-0.36 to 0.42)
5-year	-0.19 (-0.51 to 0.14)	-0.28 (-0.52 to -0.04)	-0.09 (-0.50 to 0.31)
Mean Ry, °			
1-year	0.13 (-0.06 to 0.32)	0.08 (-0.06 to 0.21)	-0.05 (-0.29 to 0.18)
2-year	0.12 (-0.09 to 0.34)	0.09 (-0.06 to 0.24)	-0.03 (-0.29 to 0.23)
5-year	0.10 (-0.12 to 0.32)	0.11 (-0.05 to 0.27)	0.01 (-0.26 to 0.28)
Mean Rz, °			
1-year	-0.10 (-0.28 to 0.09)	-0.07 (-0.19 to 0.06)	0.03 (-0.20 to 0.25)
2-year	-0.09 (-0.27 to 0.10)	-0.05 (-0.18 to 0.07)	0.03 (-0.19 to 0.26)
5-year	-0.07 (-0.27 to 0.12)	-0.08 (-0.22 to 0.06)	-0.01 (-0.25 to 0.23)
Mean TR, °			
1-year	1.48 (1.22 to 1.73)	1.37 (1.19 to 1.54)	-0.11 (-0.42 to 0.20)
2-year	1.48 (1.22 to 1.74)	1.52 (1.34 to 1.70)	0.04 (-0.29 to 0.36)
5-year	1.46 (1.18 to 1.73)	1.53 (1.32 to 1.73)	0.07 (-0.28 to 0.41)

Tx: X-translation, Ty: Y-translation, Tz: Z-translation, Rx: X-rotation, Ry: Y-rotation, Rz: Z-rotation, TT: Total-translation, TR: Total rotation. OA: osteoarthritis, RSA: radiostereometric analysis.

2

Supplementary Table S3b. Mean values in moderate- and severe OA groups across cemented and cementless implants with 95% confidence interval (CI)

Follow-up	Moderate OA mm (CI)	Severe OA mm (CI)	Mean difference mm (CI)
**Cementless UKA**			
Mean Tx, mm			
1-year	0.08 (0.04 to 0.12)	0.10 (0.07 to 0.13)	0.03 (-0.02 to 0.08)
2-year	0.13 (0.09 to 0.17)	0.14 (0.11 to 0.17)	0.01 (-0.04 to 0.07)
5-year	0.22 (0.18 to 0.26)	0.22 (0.19 to 0.25)	0.00 (-0.06 to 0.06)
Mean Ty, mm			
1-year	-0.30 (-0.37 to -0.23)	-0.31 (-0.36 to -0.25)	-0.01 (-0.10 to 0.08)
2-year	-0.28 (-0.35 to -0.21)	-0.30 (-0.36 to -0.25)	-0.02 (-0.11 to 0.07)
5-year	-0.27 (-0.35 to -0.20)	-0.29 (-0.35 to -0.23)	-0.01 (-0.11 to 0.08)
Mean Tz, mm			
1-year	-0.15 (-0.20 to -0.09)	-0.08 (-0.18 to -0.03)	0.07 (0.00 to 0.14)
2-year	-0.15 (-0.21 to -0.10)	-0.05 (-0.09 to -0.00)	0.11 (0.03 to 0.18)
5-year	-0.17 (-0.22 to -0.11)	-0.09 (-0.14 to -0.05)	0.07 (-0.00 to 0.15)
Mean TT, mm			
1-year	0.57 (0.50 to 0.64)	0.51 (0.45 to 0.56)	-0.06 (-0.15 to 0.03)
2-year	0.57 (0.50 to 0.65)	0.54 (0.49 to 0.59)	-0.03 (-0.12 to 0.05)
5-year	0.64 (0.57 to 0.71)	0.55 (0.50 to 0.61)	-0.09 (-0.18 to 0.00)
Mean Rx, °			
1-year	-0.96 (-1.21 to -0.71)	-0.80 (-0.98 to -0.60)	0.17 (-0.14 to 0.48)
2-year	-0.95 (-1.21 to -0.70)	-0.82 (-1.01 to -0.63)	0.13 (-0.18 to 0.45)
5-year	-0.99 (-1.25 to -0.73)	-0.88 (-1.08 to -0.69)	0.11 (-0.22 to 0.44)
Mean Ry, °			
1-year	-0.14 (-0.27 to -0.01)	-0.20 (-0.29 to -0.10)	-0.05 (-0.22 to 0.11)
2-year	-0.11 (-0.24 to 0.03)	-0.20 (-0.30 to -0.10)	-0.09 (-0.26 to 0.07)
5-year	-0.12 (-0.26 to 0.02)	-0.23 (-0.34 to -0.13)	-0.11 (-0.28 to 0.06)
Mean Rz, °			
1-year	0.47 (0.25 to 0.70)	0.43 (0.26 to 0.60)	-0.05 (-0.32 to 0.23)
2-year	0.31 (0.09 to 0.54)	0.30 (0.13 to 0.47)	-0.01 (-0.29 to 0.27)
5-year	0.18 (-0.04 to 0.41)	0.18 (0.13 to 0.36)	0.00 (-0.28 to 0.28)
Mean TR, °			
1-year	1.78 (1.51 to 2.06)	1.61 (1.40 to 1.82)	-0.18 (-0.53 to 0.17)
2-year	1.80 (1.52 to 2.08)	1.65 (1.44 to 1.86)	-0.14 (-0.49 to 0.21)
5-year	1.83 (1.54 to 2.11)	1.69 (1.47 to 1.90)	-0.14 (-0.50 to 0.22)

For abbreviations, see Table S3a.

Supplementary Table S3c. Mean values in moderate- and severe OA groups across cemented and cementless implants with 95% confidence interval (CI)

Follow-up	Moderate OA mm (CI)	Severe OA mm (CI)	Mean difference mm (CI)
**Cemented TKA**			
Mean Tx, mm			
1-year	-0.05 (-0.11 to 0.18)	-0.03 (-0.07 to 0.02)	0.02 (-0.06 to 0.10)
2-year	-0.05 (-0.11 to 0.02)	-0.04 (-0.09 to 0.01)	0.01 (-0.07 to 0.09)
5-year	-0.02 (-0.09 to 0.05)	-0.06 (-0.11 to -0.01)	-0.04 (-0.13 to 0.05)
Mean Ty, mm			
1-year	0.12 (0.08 to 0.16)	0.10 (0.07 to 0.14)	-0.02 (-0.07 to 0.04)
2-year	0.16 (0.12 to 0.21)	0.12 (0.08 to 0.15)	-0.04 (-0.10 to 0.01)
5-year	0.20 (0.14 to 0.25)	0.14 (0.10 to 0.19)	-0.05 (-0.12 to 0.02)
Mean Tz, mm			
1-year	-0.02 (-0.13 to 0.10)	-0.11 (-0.19 to -0.03)	-0.09 (-0.23 to 0.05)
2-year	0.01 (-0.12 to 0.13)	-0.08 (-0.17 to 0.01)	-0.08 (-0.24 to 0.07)
5-year	0.01 (-0.14 to 0.15)	-0.07 (-0.18 to 0.04)	-0.08 (-0.26 to 0.10)
Mean TT, mm			
1-year	0.45 (0.37 to 0.53)	0.53 (0.47 to 0.59)	0.08 (-0.02 to 0.18)
2-year	0.48 (0.39 to 0.57)	0.56 (0.50 to 0.63)	0.8 (-0.03 to 0.19)
5-year	0.52 (0.41 to 0.63)	0.61 (0.54 to 0.69)	0.09 (-0.04 to 0.23)
Mean Rx, °			
1-year	0.06 (-0.12 to 0.24)	-0.11 (-0.24 to 0.019)	-0.17 (-0.39 to 0.05)
2-year	0.05 (-0.14 to 0.24)	-0.13 (-0.27 to 0.01)	-0.18 (-0.42 to 0.05)
5-year	-0.02 (-0.23 to 0.20)	-0.24 (-0.40 to -0.08)	-0.23 (-0.50 to 0.04)
Mean Ry, °			
1-year	0.05 (-0.21 to 0.30)	-0.14 (-0.33 to 0.04)	-0.19 (-0.51 to 0.13)
2-year	-0.06 (-0.031 to 0.20)	-0.23 (-0.42 to -0.05)	-0.18 (-0.49 to 0.13)
5-year	-0.07 (-0.37 to 0.23)	0.02 (-0.20 to 0.24)	0.09 (-0.28 to 0.46)
Mean Rz, °			
1-year	0.03 (-0.09 to 0.14)	0.01 (-0.07 to 0.09)	-0.02 (-0.16 to 0.12)
2-year	0.03 (-0.09 to 0.16)	-0.01 (-0.10 to 0.08)	-0.04 (-0.20 to 0.11)
5-year	-0.02 (-0.17 to -0.13)	-0.00 (-0.11 to 0.11)	0.02 (-0.17 to 0.21)
Mean TR, °			
1-year	0.95 (0.73 to 1.17)	1.17 (1.01 to 1.33)	0.22 (-0.05 to 0.50)
2-year	1.02 (0.80 to 1.24)	1.19 (1.03 to 1.35)	0.17 (-0.10 to 0.44)
5-year	1.17 (0.92 to 1.42)	1.33 (1.15 to 1.52)	0.16 (-0.15 to 0.48)

For abbreviations, see Table S3a.

Supplementary Table S3d. Mean values in moderate- and severe OA groups across cemented and cementless implants with 95% confidence interval (CI)

Follow-up	Moderate OA mm (CI)	Severe OA mm (CI)	Mean difference mm (CI)
**Cemented UKA**			
Mean Tx, mm			
1-year	0.10 (0.05 to 0.15)	0.12 (0.04 to 0.19)	0.02 (-0.07 to 0.11)
2-year	0.13 (0.07 to 0.18)	0.14 (0.05 to 0.22)	0.01 (-0.09 to 0.11)
5-year	0.17 (0.09 to 0.24)	0.17 (0.06 to 0.28)	0.00 (-0.13 to 0.13)
Mean Ty, mm			
1-year	0.09 (0.03 to 0.15)	0.05 (-0.04 to 0.14)	-0.04 (-0.14 to 0.07)
2-year	0.12 (0.05 to 0.18)	0.06 (-0.04 to 0.16)	-0.05 (-0.17 to 0.07)
5-year	0.13 (0.06 to 0.20)	0.08 (-0.03 to 0.19)	-0.05 (-0.18 to 0.08)
Mean Tz, mm			
1-year	-0.06 (-0.11 to -0.00)	-0.01 (-0.09 to 0.07)	0.04 (-0.06 to 0.15)
2-year	-0.07 (-0.13 to -0.02)	-0.04 (-0.12 to 0.04)	0.03 (-0.07 to 0.13)
5-year	-0.02 (-0.10 to 0.05)	-0.06 (-0.18 to 0.06)	-0.04 (-0.18 to 0.11)
Mean TT, mm			
1-year	0.31 (0.25 to 0.38)	0.34 (0.24 to 0.43)	0.02 (-0.10 to 0.14)
2-year	0.34 (0.27 to 0.41)	0.39 (0.28 to 0.50)	0.05 (-0.08 to 0.18)
5-year	0.42 (0.33 to 0.51)	0.45 (0.31 to 0.59)	0.03 (-0.14 to 0.20)
Mean Rx, °			
1-year	-0.24 (-0.35 to -0.13)	-0.24 (-0.40 to -0.07)	0.01 (-0.20 to 0.21)
2-year	-0.29 (-0.43 to -0.16)	-0.37 (-0.58 to -0.17)	-0.08 (-0.33 to 0.17)
5-year	-0.30 (-0.46 to -0.15)	-0.39 (-0.62 to -0.15)	-0.08 (-0.37 to 0.20)
Mean Ry, °			
1-year	0.04 (-0.05 to 0.13)	0.01 (-0.13 to 0.15)	-0.02 (-0.19 to 0.14)
2-year	0.02 (-0.07 to 0.11)	0.02 (-0.12 to 0.16)	0.00 (-0.17 to 0.17)
5-year	-0.03 (-0.15 to 0.08)	0.02 (-0.16 to 0.19)	0.05 (-0.16 to 0.26)
Mean Rz, °			
1-year	-0.22 (-0.35 to -0.09)	-0.25 (-0.44 to -0.05)	-0.03 (-0.26 to 0.21)
2-year	-0.28 (-0.42 to -0.13)	-0.28 (-0.49 to -0.06)	0.00 (-0.26 to 0.27)
5-year	-0.36 (-0.54 to -0.18)	-0.41 (-0.69 to -0.13)	-0.05 (-0.39 to 0.29)
Mean TR, °			
1-year	0.75 (0.63 to 0.87)	0.75 (0.57 to 0.93)	-0.00 (-0.22 to 0.22)
2-year	0.84 (0.70 to 0.98)	0.84 (0.63 to 1.06)	0.01 (-0.26 to 0.27)
5-year	0.99 (0.81 to 1.17)	0.94 (0.67 to 1.22)	-0.05 (-0.38 to 0.29)

For abbreviations, see Table S3a.

Supplementary Table S4. Comparison of MTPM between OA groups presented in millimeters (mm), stratified by implant type

Follow-up	Moderate OA [a]<br>Mean (SE)	Severe OA [a]<br>Mean (SE)	Difference[b]<br>Mean difference (CI)
Oxford UKA, cemented			
1-year	0.56 (0.05)	0.58 (0.08)	0.03 (–0.16 to 0.22)
2-year	0.62 (0.06)	0.68 (0.09)	0.07 (–0.15 to 0.29)
5-year	0.73 (0.07)	0.78 (0.11)	0.05 (–0.21 to 0.31)
Oxford UKA, cementless			
1-year	1.22 (0.09)	1.09 (0.06)	–0.13 (–0.34 to 0.09)
2-year	1.22 (0.09)	1.13 (0.07)	–0.09 (–0.30 to 1.12)
5-year	1.28 (0.09)	1.16 (0.07)	–0.13 (–0.34 to –0.09)
NexGen TRAB MET CR Monoblock, cementless			
1-year	1.21 (0.10)	1.21 (0.07)	–0.00 (–0.24 to 0.24)
2-year	1.22 (0.10)	1.31 (0.07)	0.09 (–0.16 to 0.34)
5-year	1.21 (0.12)	1.36 (0.09)	0.15 (–0.14 to 0.44)
NexGen Stemmed Precoat, cemented			
1-year	0.98 (0.11)	1.14 (0.08)	0.17 (–0.11 to 0.44)
2-year	1.05 (0.12)	1.20 (0.09)	0.15 (–0.14 to 0.43)
5-year	1.16 (0.13)	1.32 (0.10)	0.16 (–0.17 to 0.49)
Triathlon Primary TKA, cemented			
1-year	0.61 (0.16)	0.97 (0.10)	0.22 (–0.09 to 0.53)
2-year	0.80 (0.11)	0.95 (0.08)	0.16 (–0.11 to 0.43)
5-year	1.06 (0.21)	1.17 (0.14)	–0.06 (–0.53 to 0.40)
Triathlon Primary TKA, cementless			
1-year	0.98 (0.22)	0.84 (0.11)	–0.14 (–0.63 to 0.35)
2-year	0.93 (0.25)	0.93 (0.12)	0.00 (–0.54 to 0.55)
5-year [c]	--	--	--
Vanguard TKA, cemented			
1-year	0.79 (0.57)	1.39 (0.36)	0.60 (–0.75 to 1.94)
2-year	0.80 (0.63)	1.48 (0.40)	0.68 (–0.79 to 2.15)
5-year	0.86 (0.59)	1.60 (0.37)	0.74 (–0.64 to 2.12)

Moderate OA: KL score of 1-3
Severe OA: KL score of 4
MTPM: maximum total point motion, TKA: total knee arthroplasty, UKA: unicompartmental knee arthroplasty, SE: Standard Error, CI: 95% confidence interval, BMI: body mass index, OA: osteoarthritis.
[a] Estimated mean MTPM in OA groups using mixed-effect model, BMI adjusted.
[b] Mean MTPM difference between moderate and severe OA groups at each follow-up.
[c] 5-year data pertaining to the Triathlon Primary TKA (cementless) group could not be calculated by Stata, due to a too small sample size.

Supplementary Table S5. Risk ratios (RR) for continuous migration in severe osteoarthritis (OA) group compared with moderate OA group, stratified by implant type and adjusted for age sex and BMI

Implant type	n	RR (CI)
Oxford UKA, cemented	103	2.18 (1.20–3.95)
Oxford UKA, cementless	341	1.41 (0.79–2.51)
NexGen TRAB MET CR Monoblock, cementless	210	2.13 (1.17–3.87)
NexGen Stemmed Precoat, cemented	137	1.02 (0.59–1.76)
Triathlon Primary TKA, cemented		-- [a]
Triathlon Primary TKA, cementless	15	0.61 (0.06–6.19)
Vanguard TKA, cemented	13	0.91 (0.1–8.69)

TKA: total knee arthroplasty, UKA: unicompartmental knee arthroplasty, BMI: body mass index, CI: 95% confidence interval.
[a] Data pertaining to the Triathlon Primary TKA (cemented) group could not be calculated by Stata, due to a too small sample size.

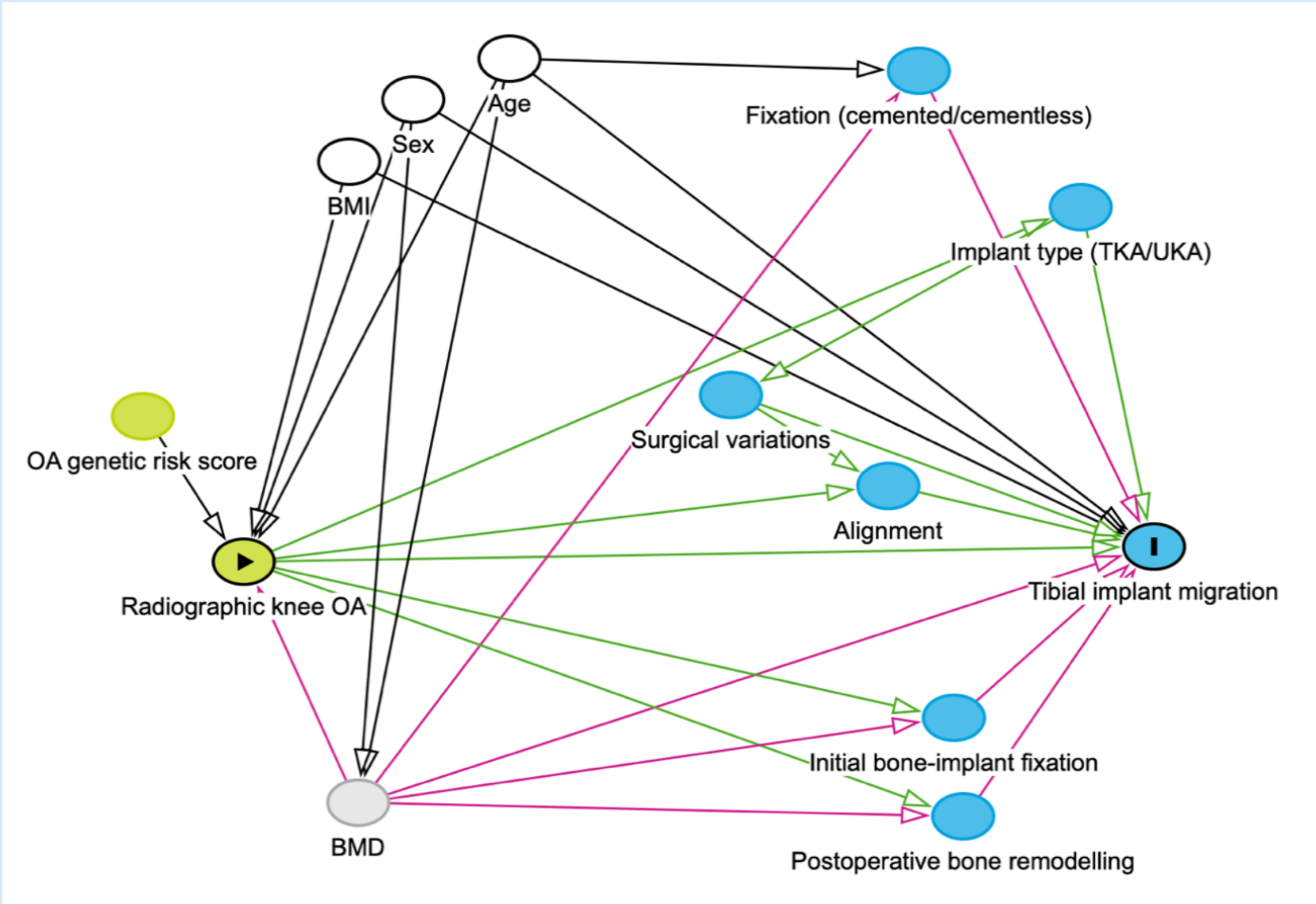

Radiographic knee OA (osteoarthritis): Exposure.
Tibial implant migration: Outcome.
Age, sex, BMI: Adjusted confounders (observed).
BMD (Bone mineral density): Unadjusted confounder (unobserved).
Initial bone-implant fixation, postoperative bone remodelling: Considered part of the causal pathway between exposure and outcome (unobserved).
Genetic risk score: Affects the exposure (unobserved).
Alignment: Considered part of the causal pathway between OA and migration and potentially influenced by surgical variations (unobserved).
Surgical variations: Considered part of the causal pathway between implant type (TKA/UKA) and migration (unobserved).
Implant type (TKA (total knee arthroplasty), UKA (unicompartmental knee arthroplasty): Considered part of the causal pathway between OA and tibial implant migration, and to potentially modify the association. Analyses were therefore stratified by these factors.
Fixation method (cemented vs cementless): Considered a part of the causal pathway between BMD and tibial implant migration. To avoid inappropriate covariate adjustment and as fixation may modify the association between OA and implant migration, analyses were stratified by fixation method.

Supplementary Figure S2. Directed acyclic graph (DAG) of the association between knee osteoarthritis severity and tibial implant migration, including hypothesized confounders, mediators, and colliders.
